# Factors Influencing Medication Adherence Among Adults Living with Diabetes and Comorbidities: a Qualitative Systematic Review

**DOI:** 10.1007/s11892-023-01532-0

**Published:** 2023-12-19

**Authors:** Kendall Gow, Amineh Rashidi, Lisa Whithead

**Affiliations:** 1 Hollywood Private Hospital, 115 Monash Ave, Nedlands, WA 6009 Australia; 2 School of Nursing and Midwifery, Edith Cowan University Joondalup Campus, Joondalup, WA 6027 Australia

**Keywords:** Diabetes mellitus, Medication compliance, Comorbidity, Medication adherence, Systemic review, Qualitative study, Adults, Chronic diseases, Patient compliance, Diabetic

## Abstract

**Purpose of review:**

Medication adherence plays an important role in improving health outcomes related to diabetes and comorbidity. The potential factors influencing medication adherence and how they contribute to health behaviors have not been synthesized to date. This review synthesized qualitative studies that identified factors influencing medication adherence among adults living with diabetes and comorbidity.

**Recent findings:**

Twenty-eight findings were extracted and synthesized into four themes: perceived support, lack of knowledge, medication issues, and the importance of routine. The findings highlight the factors that support medication adherence and areas that can be targeted to support and promote medication adherence. The findings also support the potential role of healthcare providers in supporting people living with diabetes and comorbidity to adhere to and maintain medication regimes.

**Summary:**

Several factors were identified that are amenable to intervention within the clinical practice setting and have the potential to enhance medication adherence and improve health outcomes for people living with diabetes and comorbidities. The development of acceptable and effective interventions could have a positive effect on medication adherence and health outcomes.

**Supplementary Information:**

The online version contains supplementary material available at 10.1007/s11892-023-01532-0.

## Introduction

Diabetes mellitus is a condition that affects adults globally [4] across all socioeconomic backgrounds and can lead to macro and micro vascular changes when glucose levels are not maintained within the recommended levels [1–3]. The number of people diagnosed with diabetes is on the rise, with a current estimate of 420 million people worldwide, projected to increase by 2040 to 640 million people [2, 4]. Diabetes is also linked with high morbidity and mortality, leading to 6.7 million deaths in 2021 [5]. Consequently, higher healthcare expenditure on management of diabetes and its complications are inevitable, contributing to global costs of $850 (USD) billion in 2017, and this figure is expected to increase by 7% to $958 billion in 2045 [6]. Diabetes-related complications predict poor outcomes for individuals leading to reduced quality of life, increased risk of developing other chronic diseases, and high burden of healthcare costs [7]. Therefore, total treatment adherence is essential to protect the health of individuals with diabetes [2] and reduces growing healthcare expenditure [8•].

Self-care plays an essential role in maintaining blood glucose levels (BGL) within the target range [9], medication adherence, monitoring of BGLs, exercise, and maintaining routine appointments with healthcare providers all contribute to health outcomes [10, 11]. Self-care management has been correlated with positive outcomes including optimal glucose control and the reduction of complications [12]. Adherence is defined as “the extent to which a person’s behavior taking medication, following a diet, and executing lifestyle changes, corresponds with agreed recommendations from a health care provider”(13, p.18). Pharmacological therapy is often a necessary part of chronic disease management [14]. Medication adherence becomes increasingly challenging when the complexity of managing comorbid diseases is also required [15]. Adherence is influenced by many factors like treatment complexity, duration of treatment, health care system (patient and healthcare provider relationship), adverse reactions to medications, interruption in routines, and family support [8•].

Despite what we know about the importance and benefits of treatment adherence, adherence to diabetes medication varies widely, between 36 and 90% [8•]. A recent meta-analysis included cross-sectional studies that focused only on factors associated with medication adherence and reported non-adherence to anti-diabetic medications to be 43.4% [16••]. The most recent meta-analysis that included quantitative studies on patients with diabetes regardless of age and gender, reported the rate of adherence to medication among adults living with diabetes to be 54% [17••]. It is suggested that adherence rates are higher when a person has an acute condition versus a chronic condition with adherence reducing dramatically after 6 months [18]. Low adherence to medication can lead to the exacerbation of symptoms and complications [8, 15]; for example, higher risks of macrovascular and microvascular disease among patients who were non-adherent to medication [8, 15]. Healthcare professionals also contribute to adherence and a multidisciplinary approach to patient care is recommended [8•]. The role of healthcare professionals and their impact on medication adherence is essential to explore where healthcare professionals are involved in decision-making, prescribing, treating, support, lifestyle modification, and education [8•]. A synthesis of qualitative findings exploring factors that influence medication adherence of patients with diabetes and comorbidity has not been undertaken. With the rise in multimorbidity understanding factors that influence adherence beyond the diagnosis of single disease is important. This review synthesized qualitative data on factors that influence medication adherence among patients living with diabetes and comorbidity.

## Methods

A review protocol was prepared to guide the review process and registered with Prospero (CRD42022380751). The systematic review has been reported according to the Preferred Reporting Items for Systematic Review and Meta‐Analyses: the PRISMA statement [19].

### Search Strategy

The searches were conducted across six electronic databases: Medline, PsycINFO, CINAHL, Embase, Scopus, and Cochrane. The first author searched the databases for peer-reviewed articles in English from January 2001 to October 2023. We also set up alerts for our searches in different databases to receive email notifications of new records added. Medical subject headings (MeSH) and keywords relevant to diabetes mellitus, medications/treatment adherence, and comorbidity were used in the search strategy. Lastly, the reference lists of the identified studies were reviewed to identify additional articles. Advice from a subject librarian was sought in the building of the search strategies (Appendix 1).

### Study Selection and Inclusion Criteria

All articles were imported into EndNote X9, and duplicate articles were removed. Two independent reviewers screened the titles and abstracts of the full-text articles against the inclusion criteria.

#### Inclusion Criteria

- Qualitative studies using methodologies that included phenomenology, grounded theory, ethnography, action research, and qualitative description.
- Studies conducted in community and healthcare settings.
- Studies of adults aged 18 years and over, diagnosed with diabetes and comorbidity.
- Studies published in English between 2001 and 2022 to align with the start of the discourse on medication adherence in 2001.

#### Exclusion Criteria

- Studies that included participants under 18 years old, did not define participants as living with diabetes and comorbidity.
- Studies did not include data related to medication adherence and did not include illustrative quotations.

### Quality Appraisal and Data Extraction

Two independent reviewers undertook methodological quality appraisal. The studies were assessed using the JBI Critical Appraisal Checklist for Qualitative Research. All reviewers discussed and resolved any disagreements that arose between them. A standardized data extraction tool was used to extract qualitative data from the included studies. Extracted data included population, setting, geographical location, study design, and findings.

### Data Synthesis

The JBI approach to meta-aggregation was used to synthesize qualitative data. Findings from the included studies were aggregated to create a set of categories. The extracted findings were rated according to three levels: unequivocal, credible, and unsupported. “Unequivocal: findings accompanied by an illustration beyond a reasonable doubt, therefore, not open to challenge; credible: findings accompanied by the illustrations that are plausible and inferred from the date, therefore, open to challenge; and unsupported: findings not supported by the data.” The first reviewer grouped unequivocal or credible findings into categories according to their similarity in meaning and concepts. After that, these categories were aggregated by commonality into synthesized categories. The review team discussed the categories, synthesized findings, and refined them to confirm agreement before finalization.

### Assessing Confidence

The final synthesized findings were graded according to the ConQual approach (Appendix 2). This approach considered the dependability and credibility of the findings. Five questions (Q2, Q3, Q4, Q6, and Q7) of the JBI Critical Appraisal Checklist for Qualitative Research assessed dependability. A grade of 4 or above out of five indicates a high level of dependability, whereas a grade of 3 or below indicates a moderate level of dependability. In this review, four studies [20–23] and three studies [24–26] received high and moderate dependability ratings, respectively. Credibility was obtained by establishing the congruency between the author’s interpretation and supporting data. The findings in this review were unequivocal and credible; hence, the overall credibility of the findings was downgraded from a high level to a moderate level.

## Results

### Study Inclusion

A total of 57 articles were identified through the database and manual searching. Duplicate articles were removed (*n* = 40) and 17 studies were assessed at the title and abstract level. Two independent reviewers conducted the screening process. Thirteen articles were retained for full-text screening; seven were retained for quality appraisal and were included in the synthesis. Appendix 3 illustrates the stages of the study selection process.

### Methodological Quality of Included Studies

The methodological quality assessment of the 7 included studies was conducted by two independent reviewers (Appendix 4). All included studies stated the research methodology and the research question or objectives and used appropriate data collection approaches and data analysis. Four studies [20–23] indicated the researcher’s influence on the research and vice-versa. Except for one study [24], ethical approval was reported for all included studies.

### Characteristics of Included Studies

Seven studies were included in the review. These studies were conducted either in a hospital or community setting [20–26]. Three studies were conducted in Australia [21–23], and one study each in Ghana [24], the USA [26], the UK [20], and India [25]. The sample size of studies ranged from 17 [25] to 39 [22]. Appendix 5 presents an overview of the study characteristics.

### Review Findings

Twenty-eight findings were extracted and synthesized into four themes: received support, lack of knowledge, medication issues, and the importance of routine.

#### Perceived Support

Findings from four studies [21, 22, 24, 26] contributed to this category. Family and healthcare providers were perceived as important sources of support in encouraging medication adherence. The family provided emotional support: “I don’t think I’d be alive truly if it weren’t for my husband… the wonderful part about that is it’s great to have that support when you don’t feel good” (26, p.22–23). Participants believed that having to take multiple medications can be overwhelming and family members played an important role in supporting medication adherence: “As you know they (my medicines) are laid out for me—left to my own devices, I don’t know how confident I’d be. I fob it off—my wife puts them out” (21, p.8). Support from healthcare providers clear communication and understanding of the importance of adhering to medication were described: “I think a doctor who is upfront and honest and open with you and creates an atmosphere that you feel very comfortable bringing up anything—personal problems at home, wherever—because all that affects the medication. I remember asking Dr. at that time here, ‘Do I need all this medication? Why do I need all this medication? Why do I need all this?’ And he said, ‘To live.’ You need all this medication to live” (26, p.23).

Participants expressed satisfaction with providers in relation to adequate explanations about the medication: “She (endocrinologist) has a fair idea of my history, they know you. It’s a lot easier, you tend to talk to them more easily. ‘‘Hang on, Charlie, you’ve got these seven or eight [tablets]. You don’t need to be on that one” (22, p.1748). However, one study [24] reported that a lack of support influenced the participants’ decision to discontinue medication. The environment and culture within the family could discourage people from taking their medication: “Where I come from, people don’t believe in scientific medications at all. So, when I returned from the hospital with the drugs, family members encouraged me to abandon them. As I started taking the drugs, my husband was not happy with that. He said, it is against the norm to take drugs. I initially took it for a joke but as he persisted I had to stop”[24].

#### The Importance of Routine

Two studies discussed the impact of routine on medication adherence [21, 23]. Having a routine was described as helpful in supporting people to remember to take their medications. Routine created a behavioral habit that could strengthen adherence. One participant commented: “When you get into a habit, you’re less likely to forget taking one” (23, p. 2113). Establishing a routinely reinforced self-discipline, which encourages repetition of the behavior or habit: “breakfast automatic—pills and blood pressure—67 years on insulin” (21, p.7). However, findings from one study [21] described how routine could negatively influence medication adherence behavior. Some participants who were on medication for a long time claimed that they deliberately did not take their prescribed medications because they became uninterested and wanted to have a break from taking medications: “I’ve been taking medicines for so long I get a bit bored with it… that’s just the way I am—for a week or so I may not take them for a day. Occasionally I don’t take my medicines—9/10ths of the time I do—I like a bit of a break from them” (21, p. 8).

#### Lack of Knowledge

Two studies [21, 22] identified that lack of knowledge could be a significant contributor to medication non-adherence; not knowing about the purpose of taking medications could discourage people from adhering to their medications. Participants stated that knowing more about the importance of good disease control earlier before developing complications could provide them with a clear expectation of disease processes: “I wasn’t aware (of the dangers) blood pressure earlier and if I was, it would have been different” (21, p.5). Not having knowledge and information about medication could inadvertently discourage people from taking medication: “I can’t read what’s on the packet, I just pick which ones I like but I couldn’t tell you which one’s doing what job”(22, p.1751).

#### Issues Related to Medication

Three studies identified issues that discouraged people from adhering to medication [20, 22, 24]. Firstly, concerns about medication side effects were perceived as an issue that discouraged people from adhering to their medications. Experiencing unpleasant side effects impacted adherence with medication plans: “What I was going through while taking the medication was unpleasant. I was feeling uncomfortable and not as normal as I used to be. Because of that I advised myself and stopped taking the medication” (24, p.9). Concerning about side effects discouraged people from taking their medications: “I decided to read about the side effects before taking the drugs and what I read scarred me. I did not make any attempt to the take medicines because I didn’t want to go through the side effects,” (24, p.9). The prioritization by condition was influential. Giving priority to medications that treated conditions seen as the most important, hence, they did not adhere to all prescribed medication appropriately: “If I stop taking medicine for diabetes, I’m not likely to go blind or lose my feet tomorrow, and I might get hit by a truck in the next 20 years. If I stop taking the medication to control my blood pressure I might have a stroke tomorrow and I don’t want to do that” (20, p.1208)*.* Polypharmacy was a recurring issue highlighted in one study [22]: “It was not pleasant definitely, when I was taking one in the morning, one in the afternoon, one in the evening and so on, it was confusing. Did I take this? Did I take it in the morning?” (22, p.1751).

## Discussion

This systematic review identified factors that influenced medication adherence among adults living with diabetes and comorbidity. Seven studies met the inclusion criteria and were included in this review. Factors, including perceived support, routine, medication issues, and lack of knowledge influenced medication adherence. Support from family and healthcare providers was important and perceived as related to medication adherence. Recent work has shown that families provided support and used cues to remind their family members about daily medication schedules [12].

Healthcare providers play an important role in supporting healthy lifestyle behavior. The interaction between health professionals and patients is one factor that is known to affect medication adherence. Successful communication between healthcare providers and patients promotes greater patient satisfaction with medical care, strengthening medication adherence [27]. However, a lack of family support was reported as a significant contributing factor to non-adherence. One study has shown that ineffective family involvement could be perceived as a barrier influencing medication adherence [28].

Based on the results of our review, medication issues were found to be related to medication adherence. Some people prioritized their medications to treat comorbid conditions because they were perceived to be more critical than the diabetes medications. This review found that concern about the experience of adverse events could lead to non-adherence. This finding is echoed in previous studies indicating that people’s willingness to continue taking medication in the face of adverse effects decreased [29].

Polypharmacy with diabetes and comorbidities where there is an increase in prescribed medication to be taken daily can cause medication non-adherence. Other studies indicated that polypharmacy could lead to nonadherence to prescribed medication simply because of the number of medications that can be missed daily [30, 31]. We found that routine was vital for medication adherence; it helped people use cues to remind them to take their medications. This finding is also in line with a study in which routine directly affected medication adherence and how it can incorporate the medication routine into an existing lifestyle [32].

We found that a lack of knowledge about medication and disease processes is linked to non-adherence. The findings of studies supported that inadequate knowledge was a barrier to medication adherence; people were more likely to be nonadherent when they had less medical knowledge about the medication they had been prescribed [33]. Likewise, a systematic review of qualitative studies reported that the main barriers to adherence to treatment were understanding of the disease, complications related to non-adherence to treatment, and lack of family support [34, 35].

The experience of adhering to medication contributed to a more positive mood, where adherence to treatment developed behaviors that motivated further positive health behaviors, for example, if taking medication meant that the person could exercise more, this then led to other positive health behaviors. Mariye et al. (2019) reported that among diabetic patients a positive attitude toward the benefits of medication was associated with improved self-care [36] and Jiang et al. (2021) that a positive attitude was associated with attendance for diabetic screening [37].

### Strengths, Limitations of the Review

The review is the first to synthesize findings of medication adherence among diabetes patients with comorbidities and made every attempt to follow JBI methodological guidelines to ensure rigor; however, this review may have some limitations. Excluding quantitative studies and including English studies in this review could limit the generalisability of the findings to a broader context and non-English speaking cultures.

## Conclusion and Implications

The review provides information and guidance to inform the development of strategies that can address factors that influence non-adherence to medication. The finding also supports the potential role of healthcare providers in diabetes care to help and support people living with diabetes and comorbidity. Every opportunity to raise awareness and provide knowledge needs to be harnessed. The use of videos or reading materials in physician and pharmacy waiting rooms creates an opportunity to reach a broad audience. The development of a brief screening tool would support the identification of patients with suboptimal medication adherence in clinical settings and create an opportunity to work with patients to improve adherence. Encouraging health care providers to identify “adherence supporters” for example a primary carer is an opportunity to work in collaboration to support medication adherence. The formation of peer patient support groups can sustain adherence. Policy makers and diabetes care providers need to consider the development of effective and sustainable models of social support to achieve optimal medication adherence in the context of lifelong disease. A better understanding of the individual processes of medication adherence in adults with diabetes and living with comorbidity is needed to provide further details plans for interventions. Further studies are required to assess the feasibility and barriers to the implementation of interventions to promote adherence, optimize the control of diabetes and comorbidity, and reduce disease progression.

### Supplementary Information

Below is the link to the electronic supplementary material.

Supplementary file1 (DOCX 32 KB)

Supplementary file2 (PDF 203 KB)

## Data Availability

Data can be made available upon request to the corresponding author.
