# Supplementary material for: Factors Influencing Medication Adherence Among Adults Living with Diabetes and Comorbidities: a Qualitative Systematic Review
Source: Curr Diab Rep. 2023 Dec 19;24(2):19–25. doi: 10.1007/s11892-023-01532-0 (PMC10798913; doi:10.1007/s11892-023-01532-0)
Supplement: Supplementary file 2 — Supplementary file2 (PDF 203 KB) [file 11892_2023_1532_MOESM2_ESM.pdf]

## Appendices

### Appendix 1: search strategy

### Medline

(“diabetes mellitus” OR (MH “diabetes mellitus”) OR (“diabete”) OR (MH “diabete”) OR (“impaired glucose tolerance”) OR (“impaired fasting glucose”) OR (“glucose intolerance”))AND ((“medication adherence”) OR (“MMtreatment adherence”) OR (“MMmedication nonadherence”) OR (“MM treatment nonadherence”) OR (“MM treatment noncompliant”) OR (“MM medication noncompliant”)) AND (“comorbidity”) OR (MH “comorbidity”) OR (“comorbiditie*”) ( MH “comorbiditie*”) OR (“co-morbid*”) OR (MH “(“co-morbid*”) (“comorbid*”) OR (MH “comorbid*”) (“chronic condition”) OR (MH “chronic condition” (“chronic disease*”) OR (MH “chronic disease*” (“chronic health”) OR (MH “chronic health”) OR (“pre-existing”) OR (MH “pre-existing”)

### CINAHL

(“diabetes mellitus” OR (MH “diabetes mellitus”) OR (“diabete”) OR (MH “diabete”) OR (“impaired glucose tolerance”) OR (“impaired fasting glucose”) OR (“glucose intolerance”))AND ((“medication adherence”) OR (“MMtreatment adherence”) OR (“MMmedication nonadherence”) OR (“MM treatment nonadherence”) OR (“MM treatment noncompliant”) OR (“MM medication noncompliant”)) AND (“comorbidity”) OR (MH “comorbidity”) OR (“comorbiditie*”) ( MH “comorbiditie*”) OR (“co-morbid*”) OR (MH “(“co-morbid*”) (“comorbid*”) OR (MH “comorbid*”) (“chronic condition”) OR (MH “chronic condition” (“chronic disease*”) OR (MH “chronic disease*” (“chronic health”) OR (MH “chronic health”) OR (“pre-existing”) OR (MH “pre-existing”)

### PsycINFO:

(“diabetes mellitus” OR (MH “diabetes mellitus”) OR (“diabete”) OR (MH “diabete”) OR (“impaired glucose tolerance”) OR (“impaired fasting glucose”) OR (“glucose intolerance”))AND ((“medication adherence”) OR (“MMtreatment adherence”) OR (“MMmedication nonadherence”) OR (“MM treatment nonadherence”) OR (“MM treatment noncompliant”) OR (“MM medication noncompliant”)) AND (“comorbidity”) OR (MH “comorbidity”) OR (“comorbiditie*”) ( MH “comorbiditie*”) OR (“co-morbid*”) OR (MH “(“co-morbid*”) (“comorbid*”) OR (MH “comorbid*”) (“chronic condition”) OR (MH “chronic condition” (“chronic disease*”) OR (MH “chronic disease*” (“chronic health”) OR (MH “chronic health”) OR (“pre-existing”) OR (MH “pre-existing”)

### Embase

('‘diabetes mellitus’/exp OR ‘diabete*/exp OR ‘diabetic*/ exp ‘impaired glucose tolerance/exp OR ‘impaired fasting glucose/ exp OR ‘glucose intolerance’) AND (‘medication adherence’/ exp OR/ exp OR ‘treatment adherence’/ exp OR ‘medication nonadherence’/ exp OR ‘treatment nonadherence’/exp OR ‘treatment noncompliant’/ exp OR ‘medication noncompliant’) AND

(‘comorbidity’/exp OR comorbiditie*/exp OR co-morbid*/exp OR comorbid*/exp OR ‘chronic condition’ /exp OR ‘chronic disease*/ exp OR ‘chronic health’/exp OR ‘pre-existing’)AND (2001:py OR 2002:py OR 2003:py OR 2004:py OR 2005:py OR 2006:py OR 2007:py OR 2008:py OR 2009:py OR 2010:py OR 2011:py OR 2012:py OR 2013:py OR 2014:py OR 2015:py OR 2016:py OR 2017:py OR 2018:py OR 2019:py OR 2020:py: OR 2021: py OR 2022) AND [humans]/lim AND [english]/lim

Appendix 2: Dependability scores for included studies

| Citation | Is there congruity between the research methodology and the research question or objectives | Is there congruity between the research methodology and the methods used to collect data? | Is there congruity between the research methodology and the representation and analysis of data? | Is there a statement locating the researcher culturally or theoretically? | Is the influence of the researcher on the research, and vice-versa, addressed? | Dependability Score |
|---|---|---|---|---|---|---|
| (Atinga et al., 2018) | Yes | Yes | Yes | Unclear | No | 3/5 Mod |
| (Pati et al., 2021) | Yes | Yes | Yes | No | No | 3/5 Mod |
| (Shirazian et al., 2016) | Yes | Yes | Yes | Unclear | Unclear | 3/5 Mod |
| (Stack et al., 2008) | Yes | Yes | Yes | Yes | Yes | 5/5 High |
| (Williams & Manias, 2013) | Yes | Yes | Yes | Unclear | Yes | 4/5 High |
| (Williams et al., 2008) | Yes | Yes | Yes | Unclear | Yes | 4/5 High |
| (Williams et al., 2009) | Yes | Yes | Yes | Unclear | Yes | 4/5 High |

Appendix 3: Study selection and PRISMA flow diagram (Moher et al., 2009)

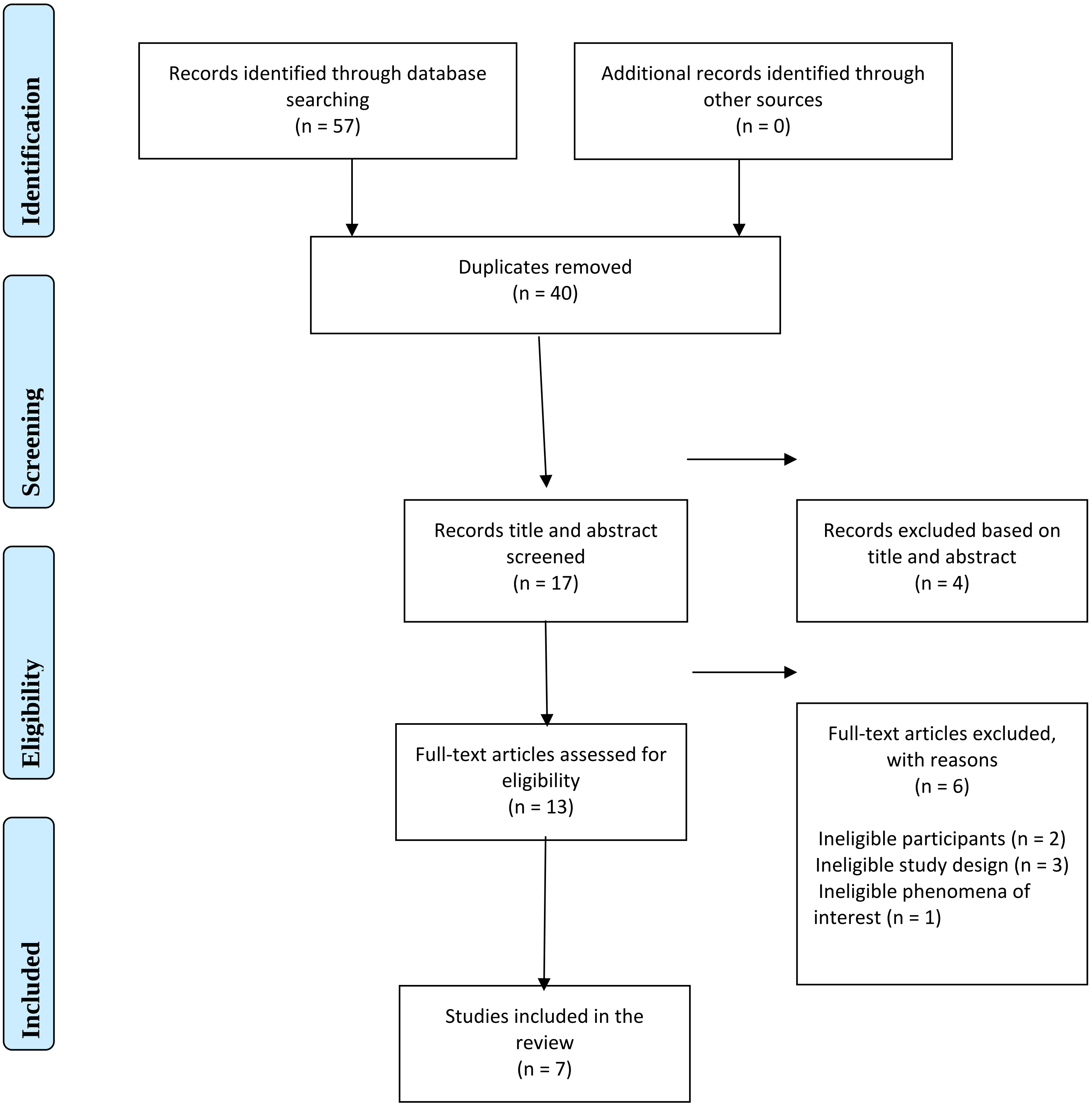

Appendix 4: Methodological quality of studies

| Citation | Q1 | Q2 | Q3 | Q4 | Q5 | Q6 | Q7 | Q8 | Q9 | Q10 | Score |
|---|---|---|---|---|---|---|---|---|---|---|---|
| (Atinga et al., 2018) | Y | Y | Y | Y | Y | U | N | Y | U | Y | 7/10 |
| (Pati et al., 2021) | Y | Y | Y | Y | Y | N | N | Y | Y | Y | 8/10 |
| (Shirazian et al., 2016) | Y | Y | Y | Y | Y | U | U | Y | Y | Y | 8/10 |
| (Stack et al., 2008) | Y | Y | Y | Y | Y | Y | Y | Y | Y | Y | 10/10 |
| (Williams & Manias, 2013) | U | Y | Y | Y | Y | U | Y | Y | Y | Y | 8/10 |
| (Williams et al., 2008) | Y | Y | Y | Y | Y | U | Y | Y | Y | Y | 9/10 |
| (Williams et al., 2009) | Y | Y | Y | Y | Y | U | Y | Y | Y | Y | 9/10 |

Y, yes; N, no; U, unclear.
Questions:
1. Is there congruity between the stated philosophical perspective and the research methodology?
2. Is there congruity between the research methodology and the research question or objectives?
3. Is there congruity between the research methodology and the methods used to collect data?
4. Is there congruity between the research methodology and the representation and analysis of the data?
5. Is there congruity between the research methodology and the interpretation of the results?
6. Is there a statement locating the researcher culturally or theoretically?
7. Is the influence of the researcher on the research, and vice-versa, addressed?
8. Are participants, and their voices, adequately represented?
9. Is the research ethical, according to current criteria, or for recent studies, and is there evidence of ethical approval by an appropriate body?
10. Do the conclusions drawn in the research report flow from the analysis, or interpretation, of the data?

Appendix 5: Characteristics of included studies for methodological review

| Study | Country & Study design | Setting / context/culture | Participant characteristics and Sample size | Age (years) | Sex (%) | Co-morbidity | Methods for data collection and analysis | Phenomena of interest | Description of main results |
|---|---|---|---|---|---|---|---|---|---|
| (Stack et al., 2008) | United Kingdom Qualitative study | Two general practices in the city | n = 19, 10 female and 9 males. | Mean age 65.3 years and age range 41-82 years | 53% female 47% male | Type 2 diabetes mellitus (T2DM) and cardiovascular disease (CVD) | The study aimed to explore people's beliefs with comorbid T2DM and CVD taking multiple medications. Patients had to be taking two or more medicines for T2DM with complications of CVD. Between May and October 2006. Semi structured interviews. Data saturation reached. The interviews analysed using grounded theory modified framework. | Explore perceptions held by people managing comorbid type 2 diabetes and CVD towards taking multiple medications | Participant scepticism if prescribed more medications, especially CVD medicines. T2DM medications were perceived as more important than CVD medications. Lower status given to CVD medications as lifestyle changes were thought to reduce CVD risk factors. Participants often ceased lipid lowering medications as a test of necessity as they were often thought to be the least important. |
| (Williams et al., 2008) | Australia | Combined diabetes and | Consumers: n = 23, 15 male and 8 females. | Consumers: | Consumers: | Diabetes and chronic | A descriptive, exploration to determine the | Understand the barriers of medication | Consumers not convinced about the safety, need and effectiveness for all |

| | | | | | | | | | |
|---|---|---|---|---|---|---|---|---|---|
| | Qualitative study | nephrology department | Health professionals: n = 16, 9 female and 7 males. | Age range 30-76 years and mean age 59.3 years<br><br>Health professionals: Mean age 43 years | 65% male 35% female<br><br>Health professionals: 56% female 44% male | kidney disease (CKD) | barriers of adhering to many recommended medications for patients with comorbid diabetes and CKD. Participants had to speak English, have diabetes and CKD, mentally competent and over 18 years of age. Exclusion if on patient on a wait list for dialysis, pregnant, cancer or have a mental health illness not medicated. Structured interviews. Focus groups undertaken with health providers to help clarify issues with being a part of the continuum | adherence from the perspectives of patients living in the community with diabetes and kidney disease and the healthcare professionals | medications. Health professionals had more focus on the need to adhere to medications and that the risk of potential adverse reactions to medications was overrated. Problems with continuity of care and accessing medications contributed to unintended medication non-adherence. Persistence of taking medications was hard for consumers. The healthcare and patient relationships were affected by healthcare inadequacies. |

| | | | | | | | | | |
|---|---|---|---|---|---|---|---|---|---|
| | | | | | | | of care and individual interviews conducted with participants to understand the complexity of managing multiple chronic conditions. Data analysed according to a medication adherence model and audiotaped and transcribed verbatim. | | |
| (Williams et al., 2009) | Australia Qualitative study | Nephrology department of a large metropolitan hospital in Melbourne | n = 23, 15 males and 8 females | Mean age 59.3 years | 65% male 35% female | Diabetes and CKD | A descriptive, exploratory design used. Review perceptions of people with diabetes and kidney disease of how irrational thought contributes to medication adherence. Study conducted between May | Explore participant medication taking from the initial prescription to when they would usually take their medicines during the day. | Patients, on average, had six chronic diseases including their diabetes and kidney disease. Two major themes emerged. To enhance coping with their complex health condition, denial was used, and heuristics showed that the participants had an inaccurate risk assessment or judgement of their medicines. |

| | | | | | | | | | |
|---|---|---|---|---|---|---|---|---|---|
| | | | | | | | and September 2007. Patients had to have diabetes and kidney disease, comprehend English, be cognitively intact and over 18 years of age. Exclusion if patient on a wait list for dialysis, pregnant, cancer or inadequately medicated for mental illness. Individual structured and in-depth interviews conducted, audiotaped and recruitment ended when no further themes developed from the data analysis. Framework method for analysis. | | |

| | | | | | | | | | |
|---|---|---|---|---|---|---|---|---|---|
| (Williams & Manias, 2013) | Australia Qualitative study | A nephrology and diabetes outpatient clinics of a metropolitan Hospital | n = 39, 17 females and 22 males | Mean age 68 years | 44% female and 56% male | Diabetes, CDK and hypertension | Study showing the participants responses from the motivational interviewing as part of the intervention arm of a RCT. Interviews conducted over phone at patients' home. Recruitment between 2008 and 2009. Each call to patient followed a semi-structured adherence counselling script. Notes taken during calls, patients made aware, and transcribed after each call. Information clarified. Not recorded. Thematic approach used. | Explore confidence and what motivates patients with diabetes and comorbidity to take their prescribed medication | Confidence and motivation is hindered by complex medical conditions and medicine regimes. Patients wanted more independence over their condition and would develop tactics to challenge threats made to their health. Barriers dominated the advantages of taking prescribed medication and was mostly due to polypharmacy. |

| | | | | | | | | | |
|---|---|---|---|---|---|---|---|---|---|
| (Atinga et al., 2018) | Ghana A qualitative study | Teaching hospital in Ghana's capital | n = 49, 32 patients and 17 caregivers, 28 males and 21 females | Mean age 42 years. Age range 31-57 years | 57% male and 43% female | Diabetes and hypertension | Study aimed to explore the factors that contributed to why patients with diabetes and hypertension poorly adhered to their prescribed medications. Patients that were non-adherent to their medications and subsequently were readmitted were recruited between July and December 2015. Interviews were held as discussions using inductive cues to ensure all factors covered that were not in the discussion guide. Patient consent for audio recordings | Explore the factors that may have caused medication non-adherence including family, community, personal and healthcare provider factors. | Perception that the medications were ineffective at managing their illnesses caused non-adherence. Patients with this perception would opt for herbal and spiritual medicines and healing because of accessibility, perceived efficacy and they are more affordable. Nonadherence was also influenced by work schedules, polypharmacy, poor medication education from healthcare provider, social norms, poor knowledge and experience of medications. |

| | | | | | | | | | |
|---|---|---|---|---|---|---|---|---|---|
| | | | | | | | and were transcribed and themes were guided using thematic analysis. | | |
| (Shirazian et al., 2016) | United States of America A qualitative study | Outpatient nephrology department | n = 23, 14 males and 9 females | Mean age 64 years. Age range 18-79 years | 61% male and 39% female | T2DM and CKD | Exploration of the views of patients with T2DM and CKD related to their self-management and to identify the facilitators and barriers of self-management within this population. Inclusion of English speaking adults (18-79 years), treated for T2DM, had CKD stage 2-5 and were prescribed at least one antihypertensive. Exclusion of patients if they | Explore self-management barriers and facilitators of patients with T2DM and CKD | Findings found that emotional responses to health status, family dynamics and how that impacts or creates a burden on self-management. Participants found no improvement in health just maintenance. Emotional responses to current health status and future of potential dialysis – regret of past non-adherence to medication regimes. Frustration of the number of healthcare professionals involved in their care, number of medications they had to take and restriction of dietary intake. Motivation of current health status to make improvements – in order to reduce medication intake.<br>Family/partners seen as barriers to support health |

| | | | | | | | | | |
|---|---|---|---|---|---|---|---|---|---|
| | | | | | | | did not have T2DM and CKD or had another medical condition. Semi-structured questionnaire in focus groups. Audiotaped sessions, transcribed. Thematic analysis. Data checked before initial coding. Multiple reviews of the transcripts to verify themes. Triangulation of data – member checking and three participants | | maintenance like food intake.<br>Family provide support however controlling behaviour could undermine care.<br>Communication from health professionals not appropriate for self-care – leading to distrust of their healthcare providers.<br>Appointments (multiple) took a lot of time and energy. Feelings of overwhelm with management. Anguish over polypharmacy. Side effects of medication.<br>Lack of understanding of medications. Adhering to dietary modifications or instructions was challenging. Barriers to physical activity included physical restrictions and lack of motivation |
| (Pati et al., 2021) | India A qualitative study | Urban health centres (17) | n = 17, 10 female and 7 males | Mean age 40 years. Age range | 59% female and 41% female | Diabetes and comorbidities | Study to explore the perceived obstacles and enablers of patients' management | Explore patients' management with comorbid diabetes by | Barriers were related to patients, health system and health providers (physicians). Major barriers to quality care was due to poor skills and |

| | | | | | | | | | |
|---|---|---|---|---|---|---|---|---|---|
| | | | | 28-61 years | | | with comorbid diabetes by primary health physicians. In-depth interviews conducted with primary care physicians and conducted in local language. The interviews were converted to English and verbatim transcription used. Thematic analysis. | primary health physicians | knowledge, overburdening and lack of communication skills. Treatment adherence among patients was facilitated by their beliefs and attitudes and socio-economic status. Barriers to optimal care were irregular supply of medicines, poor infrastructure, shortages of skilled health professionals and lack of electronic and personal records. |

Atinga, R., Yarney, L., & Minta Gavu, N. (2018). Factors influencing long-term medication non-adherence among diabetes and hypertensive patients in Ghana: A qualitative investigation. *PLoS ONE, 13*(3), 1-17. https://doi.org/10.1371/journal.pone.0193995

Moher, D., Liberati, A., Tetzlaff, J., Altman, D. G., & Group, T. P. (2009). Preferred Reporting Items for Systematic Reviews and Meta-Analyses: The PRISMA statement. *PLoS Medicine, 6*(7). https://doi.org/10.1371/journal.pmed.1000097

Pati, S., Pati, S., van den Akker, M., Schellevis, F. G., Sahoo, K. C., & Burgers, J. S. (2021, 2021/05/22). Managing diabetes mellitus with comorbidities in primary healthcare facilities in urban settings: a qualitative study among physicians in Odisha, India. *BMC Family Practice, 22*(1), 1-9. https://doi.org/10.1186/s12875-021-01454-4

Shirazian, S., Crnosija, N., Weinger, K., Jacobson, A. M., Park, J., Tanenbaum, M. L., Gonzalez, J. S., Mattana, J., & Hammock, A. C. (2016). The self-management experience of patients with type 2 diabetes and chronic kidney disease: A qualitative study. *Chronic Illness, 12*(1), 18-28. https://doi.org/10.1177/1742395315614381

Stack, R. J., Elliott, R. A., Noyce, P. R., & Bundy, C. (2008). A qualitative exploration of mulitple medicines beliefs in co-morbid diabetes and cardiovascular disease. *Diabetic Medicine, 25*(10), 1204-1210. https://doi.org/10.1111/j.1464-5491.2008.02561.x

Williams, A., & Manias, E. (2013). Exploring motivation and confidence in taking prescribed medicines in coexisting diseases: a qualitative study. *Journal of Clinical Nursing, 23*(3-4), 1-11. https://doi.org/10.1111/jocn.12171

Williams, A., Manias, E., & Walker, R. (2008). Adherence to multiple, prescribed medications in diabetic kidney disease: A qualitative study of consumers' and health professionals' perspectives. *International Journal of Nursing Studies, 45*, 1742-1756. https://doi.org/10.1016/j.ijnurstu.2008.07.002

Williams, A., Manias, E., & Walker, R. (2009). The role of irrational thought in medicine adherence: people with diabetic kidney disease. *Journal of Advance Nursing, 65*(10), 2108-2117. https://doi.org/10.1111/j.1365-2648.2009.05077.x
